# Effect of NonSurgical Periodontal Therapy on Plasma Levels of IL-17 in Chronic Periodontitis Patients with Well Controlled Type-II Diabetes Mellitus—A Clinical Study

**DOI:** 10.3390/dj6020019

**Published:** 2018-06-13

**Authors:** Vishnu Jayakumar Sunandhakumari, Arun Sadasivan, Elizabeth Koshi, Aswathy Krishna, Aneesh Alim, Aneesh Sebastian

**Affiliations:** 1 Department of Periodontics and Oral Implantology, PMS College of Dental Science and Research Centre, Trivandrum-695028, India; 2 Department of Periodontics and Oral Implantology, Sreemookambika Institute of Dental Science and Research, Tamil Nadu-629161, India; sadasivan_arun@hotmail.com (A.S.); elizabethkoshi_dr@yahoo.com (E.K.); 3 Department of Oral and Maxillofacial Surgery, Amrita School of Dental Sciences, Cochin-682041; India; aswathyavalon@gmail.com; 4 Department of Orthodontics and Dentofacial Orthopedics, PMS College of Dental Science and Research Centre, Trivandrum-695028, India; aneeshalim@gmail.com; 5 Department of Oral and Maxillofacial Surgery, PMS College of Dental Science and Research Centre, Trivandrum-695028, India; draneesh2008@gmail.com

**Keywords:** periodontitis, Diabetes mellitus, cytokines, Interleukin-17

## Abstract

For years the pathogenesis of periodontitis was under an immunological Th1/Th2 paradigm. Th1 cells are considered to afford protection against the intracellular pathogens. These cells produce the interferons (IFN) that are involved in macrophage activation, which, in turn, plays an important role in phagocytosis, complement fixation, and opsonization. Th2 cells are thought to have evolved as a form of protection against parasitic helminthes. Th17 subset of CD4Not Necessary+ T cells was identified in the year 2005, which added greater complexity to Th function and are pro inflammatory in nature. Interleukins (ILs) have the ability to alter immunological changes and they also possess the ability to regulate lymphocyte differentiation and haemopoietic stem cells, cell proliferation, and motility, which are classified as pro-inflammatory and anti-inflammatory. There are numerous studies that reported IL-17 levels associated with chronic periodontitis (CP) development. Type II diabetes mellitus (DM) is considered a risk factor for the development of periodontal diseases because the incidence, progression, and severity of periodontal diseases are more common with Type II DM than without DM. This study was aimed at evaluating whether non-surgical periodontal therapy had any effect on plasma concentrations of Interleukin-17 in systemically healthy chronic periodontitis patients and in chronic periodontitis patients with well controlled Type II Diabetes mellitus. Patients were divided into the two groups including the chronic periodontitis group (20 subjects) and the chronic periodontitis with well-controlled Type II Diabetes mellitus group (20 subjects). The Gingival Index and Plaque Index as well as the clinical Attachment Level (CAL) were taken from all the patients of two groups after evaluating fasting blood sugar, post prandial blood sugar, and the Glycated Hemoglobin Level (HbA1c). Then 5 mL blood samples were collected from each patient and plasma was separated and the IL-17 level is evaluated using the ELISA method. Then, as part of phase I periodontal therapy, scaling and root planning was performed. Patients were recalled after one month and clinical and biochemical parameters were reevaluated. Non-surgical periodontal therapy resulted in a reduction of plasma levels of IL-17 in chronic periodontitis patients with and without well controlled Type II Diabetes mellitus.

## 1. Introduction

Periodontitis, which is a chronic inflammatory infectious disease caused by the bacterial plaque biofilm colonization in the teeth, evokes host immune responses. This ultimately leads to the destruction of surrounding periodontal tissues [1]. The progression of periodontal disease depends upon interaction between microbial biofilm and host response which are primarily mediated by cytokines as well as chemokines of resident and emigrant cells in the area of inflammation [2]. Cytokines are soluble proteins secreted by cells to act as a messenger that transmits signals to other cells. They are capable of mediating aswell as to control immune and inflammatory responses and also can regulate cellular growth and differentiation [3]. Numerous cells in the human body are capable of producing cytokines such as neutrophils, dendritic cells, macrophages/monocytes lymphocytes, fibroblasts, and endothelial cells [2]. It has been proven and postulated that an appropriate production of cytokine results in protective immunity while inappropriate cytokine production results in tissue destruction and progression of disease [4]. The balance between cellular and humoral responses, for example, is highly and strongly regulated by the balance between Th1 and Th2 subsets of cells. T cells are classified into various categories such as helper T (Th) cells, cytotoxic T (Tc) cells, and regulatory T (Treg) cells are based on their function. Over the years, it has been proposed that, as stable periodontal lesions resemble a delayed type hypersensitivity lesion and progressive lesion involves large number of B cells, these lesions may be mediated by Th1 and Th2 cells, respectively. It has been shown that a strong innate response leads to a Th1 response under the influence of IL-12, IL-2, and IFN-γ while a weak innate response leads to a Th2 response under the influence of IL-4 cytokines. In a stable lesion, IFN-γ enhances the phagocytic activity of both neutrophils and macrophages and, therefore, contains the infection. In case of a poor innate immune response and minimal Il-12 production, a weak Th1 response may not contain infection. The stimulation of mast cells and the subsequent production of IL-4 helps in encouraging a Th2 response, antibody production, and also B-cell activation. If these antibodies are protective in nature and clear infection, the disease will not progress, but if they are not protective, as in the case of IgG2, the lesion will persist. This continued activation of the B-cell may result in increased amounts of IL-1 and, therefore, enhances tissue destruction [5]. It is generally agreed that control of the Th1/Th2 balance is central to the immuno-regulation of periodontal disease. This also proved that periodontal lesions, which are stable in nature are mediated by Th1 cells and a shift toward Th2 cells reflects disease progression [6].

**Figure 1:**
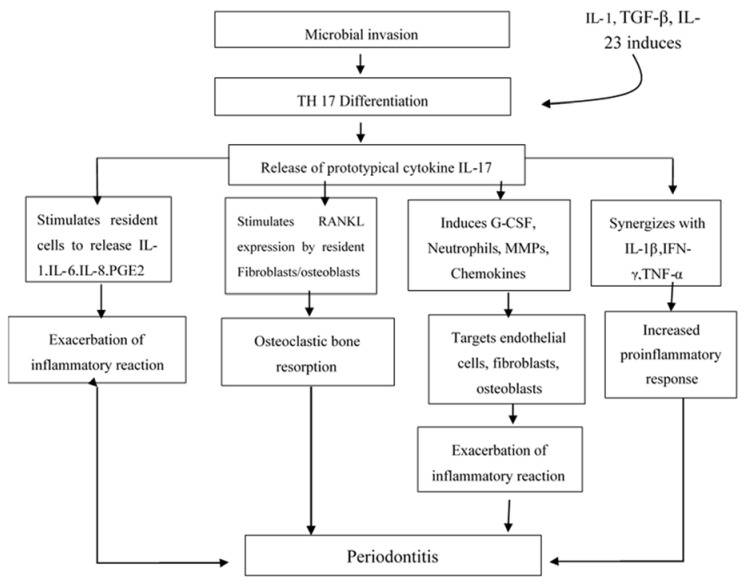
Role of IL-17 in the pathogenesis of periodontitis.

Interleukin-17 (IL-17) is a pro-inflammatory cytokine produced mainly by cells of the Th1/Th0 phenotype but not by cells of the Th2 phenotype. The IL-17 family includes IL-17A, IL-17B, IL-17C, IL-17D, IL-17E (IL-25), and IL-17F [7]. Th17 subset of CD4 T cells was identified in 2005, which uncovered the concepts associated with an inflammatory immune reaction. Th17 cells are the main CD4 T cells that produce Th17-related cytokines including interleukin (IL)-17, IL-21, IL-22, IL-6, and TNF-a. These cells have the ability to recruit macrophages and also neutrophils to participate and enhance the inflammatory immune reaction. Tissue damage seems to be intensified by uncontrolled proliferation as well as dysfunction of the Th17 cells [8]. It is proposed that induction of Th17 responses requires three distinct steps including induction, amplification, and stabilization while transforming growth factor (TGF)-β plus IL-6 as having the potential to induce Th17 cell differentiation. In addition, IL-21 amplifies the frequency of Th17 cells and IL-23 helps in stabilizing the phenotype of previously differentiated Th17 cells. The loss of any of one of the members in this pathway (IL-6, IL-21, or IL-23) severely limits the responsiveness of Th17 cells [2]. IL-17 has been shown to stimulate fibroblastic cells as well as epithelial and endothelial cells to produce IL-6, IL-8, and Prostaglandin E2 (PGE2). PGE2 enhances the permeability and dilation of the blood vessels, which results in redness and edema and has the ability to induce synthesis of matrix metalloproteinases (MMPs) by infiltrating cells such as monocytes and residents cells like fibroblasts. MMPs also causes connective tissue degradation and osteoclastic bone destruction. Both of these are classic hallmarks of the presence of periodontal disease. IL-17 also capable of inducing the receptor activator of nuclear factor kappa B ligand (RANKL) production by osteoblastsand has been has been hypothesized that those IL-17 produced in periodontal lesions, which may be involved in the modulation of Th1. IL-17 enhances inflammatory reactions with the help of gingival fibroblast-derived mediators in periodontal disease [9]. Elevated levels of IL-17, TGF-β, IL-1β, IL-6, and IL-23 messenger RNA and protein in diseased tissues as well as the presence of Th17 cells in gingiva from patients with periodontitis has been reported by Cardoso et al [10]. Moreover, the alveolar bone of diseased patients expressed abundant levels of IL-17 and the bone resorption factor RANKL in contrast to low expression in healthy controls Figure 1 [11].

The most common human endocrine disease known as Diabetes mellitus (DM) is a metabolic disorder characterized by chronic hyperglycemia and is caused by the interaction of environmental factors such as obesity, genetic susceptibility, sedentary lifestyle, and high-calorie food intake. The two main categories of diabetes mellitus are Type 1 (T1DM) and Type 2 DM (T2DM). Type 1 DM results from the autoimmune destruction of pancreatic islet cells, which eventually leads to the loss of insulin production, and T2DM, which usually occurs in adulthood and is characterized by an increase in insulin resistance associated with the inability of pancreatic beta cells to secrete sufficient amounts of insulin to compensate. DM is considered an important and independent risk factor for the development of gingivitis and periodontal disease [12]. It seems that the incidence, progression, and severity of periodontal diseases are higher in subjects with type II DM than in those without diabetes mellitus [13]. Advanced glycated end products seem to be higher in patients with diabetes mellitus. The interaction between advanced glycation end products (AGE) and its receptor for advanced glycation end products (RAGE), which is present in different types of cells, enhances the expression of pro-inflammatory cytokines. As a result of this, IL-17, which is also a pro-inflammatory cytokine, seems to increase in patients with diabetes mellitus. This evidence states that there is a chance of increased IL-17 expression in patients with diabetes mellitus as well as in periodontitis. Periodontal disease is considered a result of microvascular diabetes mellitus. There is scientific evidence that proves diabetes is one of the risk factors for developing gingivitis and periodontitis as well as the blood level glucose in this interaction. Therefore, periodontal disease is clearly a clinical manifestation associated with several systemic diseases including diabetes mellitus. Gram—anaerobic bacteria in periodontal pockets and/or gingival sulcus helps in stimulating the immune system cells and leads to a release of inflammatory mediators. These mediators get into the bloodstream, which further increase the inflammation present in diabetes mellitus and interferes with blood glucose levels’ regulation, which leads to diabetic complications [14]. Earlier it has been found that type II diabetes mellitus as a whole enhances pro-inflammatory cytokines like Interleukin-17 in periodontitis and it has also been observed that the highest levels of Interleukin-17 are seen in patients with uncontrolled Diabetes mellitus [13,15]. It has been shown that IL-17 resulted in the inhibition of uptake of glucose in vitro and also impairs glucose and insulin metabolism in metabolic syndrome and diabetes in young mice [14].

**Figure 2:**
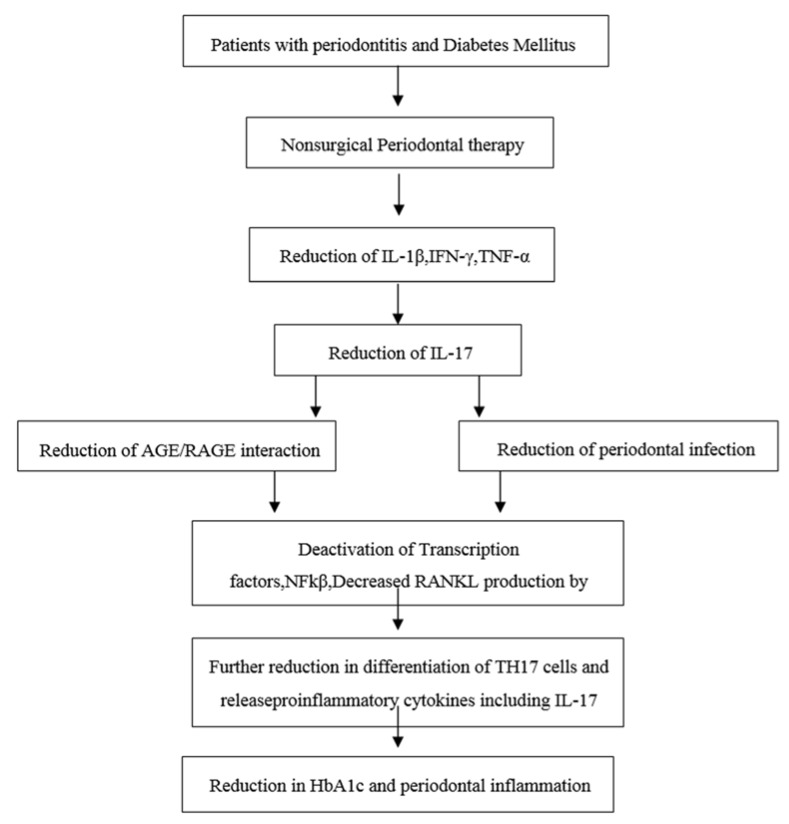
Possible role of Nonsurgical periodontal therapy in the prevention of periodontal tissue destruction and diabetes mellitus by down-regulating IL-17 levels.

In the year 2010, Kardeşler L et al. [16] evaluated the effects of periodontal treatment on clinical periodontal parameters, systemic mediators, and glycemic control in well or poorly-controlled type 2 diabetic as well as systemically healthy periodontitis patients. He showed that a poorly-controlled diabetic group exhibited significantly decreased HbA1c levels after completion of non-surgical periodontal treatment. The diabetic complications were associated with previous hyperglycemia in type 2 diabetics and any reduction in HbA1c is likely to reduce the risk of complications. HbA1c reflects an average plasma glucose level over the previous eight to 12 weeks. HbA1c is a reliable long-term marker of glycemic control. HbA1c (glycated haemoglobinA1c) occurs when the oxygen-carrying protein in red blood cells and the hemoglobin becomes bonded with glucose in the bloodstream. This bonding with glucose is called glycation. The higher a person’s blood glucose levels have been, the higher the number of red blood cells that will have become glycated, which resulted in higher levels of HbA1c. There are various studies supporting the effective role of nonsurgical periodontal treatment in the reduction of HbA1c levels in Type II DM patients. Therefore, periodontal treatment may be considered as one of the means of reducing HbA1c levels. This eventually helps in overall disease management of patients with diabetes mellitus (see Figure 2) [17].

The aim of our study is to explore the effect of non-surgical periodontal treatment on plasma levels of Interleukin-17 in systemically healthy chronic periodontitis patients and in chronic periodontitis patients with well-controlled Type II diabetes mellitus.

## 2. Materials and Methods

The study population consisted of 40 subjects aged between 30 to 65 years of age. Approval for the study was obtained from the Institutional Human Ethics Committee, SreeMookambika Institute of Dental sciences-Tamilnadu, India with Clinical trial registry of India Ref no-REF/2015/04/008775. The patients were given a detailed explanation about the study and associated treatment procedures followed by obtaining the written informed consent from subjects who agreed to participate voluntarily in this study.

Patients were considered to have chronic periodontitis when there is an attachment loss ≥5 mm in more than 30% of teeth (according to the criteria of 1999 AAP Workshop [18]) and the presence of bleeding on probing. This includes patients with a presence of more than or equal to 20 teeth. Patients were considered to be well-controlled Type 2 diabetic when presenting with fasting glucose levels in excess of 126 mg/dL and HbA1c above 6.5% and below 7% [18].

Exclusion criteria consisted of patients suffering from any systemic condition (except type II diabetes) that could affect the periodontal disease and affect the level of Plasma IL-17 levels and also smokers, alcoholics, pregnant women, and lactating mothers. Patients needing prophylactic antibiotic medication in association with periodontal probing and patients who used antibiotics or underwent periodontal treatment in the past six months were also excluded from the study. Each patient underwent periodontal probing and also charting of parameters along with fasting blood sugar and HbA1c level estimation. Based on the Gingival Index (GI), the Plaque Index (PI), the clinical attachment level (CAL), the fasting blood sugar (FBS), and HbA1c followed by categorizing of patients into two groups [18].

Group I (20 patients)-Chronic periodontitis group (FBS < 126 mg/dL and HbA1c less than 6.5%).

Group II (20 patients)-Chronic periodontitis group with well-controlled Type II diabetes mellitus (FBS > 126 mg/dL and HbA1c greater than 6.5% and less than 7).

All the enrolled study subjects were treated with non-surgical periodontal therapy (NSPT). After base line evaluation of PI, the GI, CAL, FBS, HbA1c, and plasma samples were collected and recalled after one month to evaluate the same clinical and biochemical parameters.

### 2.1. Plasma Sampling

Immediately before NSPT, the venous blood samples were collected with a 5 mL syringe using a standard venipuncture method in an EDTA-coated tube and the plasma from collected blood is separated by centrifugation at 1500 g for 5 min and plasma samples were then stored at −80 °C until it was required for biochemical analysis when they were thawed immediately before use.

### 2.2. Plasma IL-17 Analysis 

The plasma analysis of IL-17 was performed at Rajiv Gandhi center for Biotechnology, Trivandrum-India. The ELISA development kit (DIACLONE, Besancon Cedex, France) was used to analyze the concentration of IL-17 in the plasma samples. A standard volume of 10 µL was assayed after diluting the plasma samples to half its concentration. The manufacturers’ guidelines were followed for assay. The concentration of IL-17 in the plasma samples was determined by comparing the average absorbance readings of each sample with concentration in the assay standard curve. The results were expressed as pg/mL.

## 3. Statistical Analysis

The data was analyzed by using the (SPSS 16.0) version. The chi-square test was applied to find the statistical significance within and between the groups. *p* value less than 0.05 (*p* < 0.05) were considered statistically significant at 95% confidence interval. A paired T test is used for comparing the means within the groups and an unpaired T test is used to compare the means between the groups.

## 4. Results

Descriptive data for a concentration of IL-17, which includes the comparison of demographic, periodontal, and biochemical parameters within the Group I & II before and after NSPT and between the groups were outlined in Table 1, Table 2, Table 3 and Table 4.

The periodontal and biochemical parameters within the Group I & II before and after NSPT showed that there is statistically significant reduction in PI, GI, PPD, and CAL. The plasma IL-17 level was correlated with that of the periodontal parameters and was higher in Group II patients compared to that of Group I. However, there was no statistically significant reduction in case of fasting blood sugar and HbA1c in both the groups before and after NSPT.

Comparison of demographic data between the groups before and after treatment showed that the age range of Group I was 40 to 45 years of age and the age range of group II was 45 to 50 years of age. The patients in the control group was younger than in Group II. Comparison of periodontal and biochemical parameters between the groups before treatment showed that there was statistically significant higher representation of all the parameters in Group II than Group I. The mean plasma IL-17 levels in group I and group II before NSPT was 0.18 ± 0.03 pg/mL and 0.21 ± 0.04 pg/mL, respectively, and one month after NSPT, there was reduction in Plasma IL-17 levels in both the groups i.e., the baseline values were reduced to 0.16 ± 0.02 pg/mL in Group I subjects and 0.16 ± 0.03 pg/mL in Group II subjects.

The correlation of the variables shows that IL-17 is directly proportional to HbA1c level. Findings from the above table shows that non-surgical periodontal therapy leads to a decrease in probing pocket depth and an increase in the clinical attachment level. Patients with chronic periodontitis showed elevated levels of plasma IL-17.

## 5. Discussion 

Periodontal disease results from an interplay between one or more different types of virulent microorganisms, bacteria, or host factors or as a result of interaction of both these factors. These interactions, which are mediated by cytokines as a result of increased lymphocytic infiltration in the periodontal tissues and represent an important component of the immune response to bacterial toxins such as lipopolysaccharides. IL-17 is a pro-inflammatory cytokine detected in periodontal tissues, GCF, saliva, and plasma of patients with periodontal disease [2,19,20]. The interrelation between periodontal disease and diabetes mellitus has been studied and proven that the prevalence, progression, and severity of periodontal disease is higher in diabetic individuals when compared to non-diabetics [13].

The present study evaluated the plasma IL-17 levels in systemically healthy chronic periodontitis patients and in chronic periodontitis patients with well controlled Type II DM before and one month after NSPT. Plasma IL-17 levels was found to be significantly higher in patients with Type II DM before the treatment when compared to patients with chronic periodontitis alone. Numerous studies indicated that the effect of IL-17 in local inflammation might be due to its effects on inducing cells, which helps in releasing pro-inflammatory cytokines and also by recruiting polymorphonuclear neutrophils. In this study, it has been shown that there is a positive correlation between Plasma IL-17 with that of periodontal parameters and FBS and HbA1c values before and after treatment in both groups. These findings support the study done by Takahashi et al. [21], Vernal et al. in 2005 [14], and Ohayama et al. in 2009 [14], which states that patients with chronic periodontitis exhibit increased levels of IL-17 [21].

There were increased levels of plasma IL-17 in Group II patients when compared to that of Group I before and after NSPT and this may be due to the interaction between advanced glycation end products (AGE) and its receptors for advanced glycation end products (RAGE). This is present in different types of cells, which enhances the expression of IL-17. This glycation process alters the structure of proteins (extracellular matrix proteins) leading to an increase in the stiffness and resistance to a proteolytic way of digestion [13]. AGE’s have been shown to be increasingly expressed in the periodontium of diabetic patients and also associated with the deterioration of periodontitis condition while no other biochemical markers or bacterial occurrence showed a transparent relationship with that condition.

The present study showed that NSPT, which is considered as a gold standard and most effective basic therapy for the majority of periodontitis patients, produced a reduction of the Plasma IL-17 level in both the groups along with a reduction of periodontal parameters. The well-controlled diabetic group exhibited a reduction in the HbA1c level and a periodontal parameter after one month of NSPT. These findings are in contrast with the studies done by Stuart et al. in 2001 [22], Rodrique et al. in 2003 [22], Kiran M et al. in 2005 [22], and Marzieh et al. in 2013 [22]. This may be regarded as an added proof of the beneficial effect of periodontal treatment in glycemic control in Type II diabetes mellitus. The risk of diabetic complications were strongly associated with previous hyperglycemia in Type II diabetes mellitus and HbA1c reduction is likely to reduce the risk of complications it is associated with. Therefore, from the present study, it is evident that NSPT may be regarded as a measure of reducing HbA1c levels that eventually helps in the management of diabetic patients and the complications it is associated with.

Even though the present study showed the positive effects of NSPT in periodontal and biochemical parameters, there are certain limitations such as sample size and use of plasma samples, which can be rectified in further studies by selecting large samples and analyzing IL-17 levels in GCF or saliva.

## 6. Conclusions

To the best of our knowledge, this is the first study to evaluate the effect of nonsurgical periodontal therapy on the plasma IL-17 levels in systemically healthy chronic periodontitis patients and in chronic periodontitis patients with well-controlled Type II DM. Within the limits of the study, it can be concluded that the IL-17 level is positively correlated with the periodontal condition of the patient, HbA1c levels, and the fasting blood sugar level. Nonsurgical periodontal therapy plays an effective role in reducing the Th17 related cytokine (IL-17) in plasma both in systemically healthy chronic periodontitis and in chronic periodontitis with well-controlled Type 2 DM patients. It is quite evident that Type II DM increases the risk of periodontitis where there is ever increasing evidence, which shows adverse effects of periodontal diseases on diabetes mellitus onset and also in progression. Present evidence suggests educating and motivating the patients about the importance of oral hygiene, which should be a part of treatment planning and the prevention protocol. This can be achieved with a better and closer collaboration between dentists, physicians, and a referral to a periodontist, which is highly recommended after diagnosis of diabetes mellitus.

The main limitations of the study include less sample size and also it is a nonrandomized study. Furthermore, instead of using plasma for evaluating IL-17 levels, the results will be more standardized if the Gingival crevicular fluid has been used.

## Figures and Tables

**Table 1:** Comparison of periodontal and biochemical parameters within the group-I before NSPT and 1 month after NSPT. *p* Value < 0.05 was considered as statistically significant.

Observations	Before NSPT (Mean ± SD)	1 Month after NSPT (Mean ± SD)	*p* Values	Percentage Difference (%)
Fasting blood glucose level (mg/dL)	116.30 ± 6.09	113.25 ± 5.58	0.46	2.62
HbA1c (%)	5.68 ± 0.22	5.55 ± 0.26	0.31	2.31
PI	1.32 ± 0.40	0.86 ± 0.19 *	0.05	42.20
GI	1.24 ± 0.36	0.85 ± 0.25 *	0.03	37.32
PPD (mm)	2.69 ± 0.36	2.13 ± 0.27 *	0.01	23.23
CAL (mm)	3.02 ± 0.68	2.65 ± 0.59 *	0.05	13.05
IL-17 (pg/mL)	0.18 ± 0.03	0.16 ± 0.02	0.08	12.50

* *p* < 0.05 considered as statistically significant.

**Table 2:** Comparison of periodontal and biochemical parameters within the group-II before NSPT and one month after NSPT.

Observations	Before NSPT (Mean ± SD)	1 Month after NSPT (Mean ± SD)	*p* Values	Percentage Difference (%)
Fasting blood glucose level (mg/dL)	145.45 ± 6.14	142.85 ± 6.26	0.60	1.80
HbA1c (%)	6.74 ± 0.17	6.61 ± 0.20	0.33	1.95
PI	1.46 ± 0.52	0.92 ± 0.39 *	0.001	45.38
GI	1.56 ± 0.56	1.10 ± 0.36 *	0.01	34.59
PPD (mm)	2.77 ± 0.65	2.42 ± 0.65 *	0.05	13.49
CAL (mm)	3.11 ± 0.68	2.60 ± 0.72 *	0.05	17.86
IL-17 (pg/mL)	0.21 ± 0.04	0.16 ± 0.03	0.09	22.22

* *p* < 0.05 significant compared before and after treatment within the group-II.

**Table 3:** Comparison of demographic, periodontal, and biochemical parameters between the groups before NSPT.

Observations	Group-I (Mean ± SD)	Group-II (Mean ± SD)	*p* Values	Percentage Difference (%)
Age (years)	42.10 ± 8.22	46.20 ± 5.97	0.079	9.29
Fasting blood glucose level (mg/dL)	116.30 ± 6.09	145.45 ± 6.14 *	0.001	22.27
HbA1c (%)	5.68 ± 0.22	6.74 ± 0.17 *	0.001	17.07
PI	1.32 ± 0.40	1.46 ± 0.52 *	0.05	10.07
GI	1.24 ± 0.36	1.56 ± 0.56 *	0.05	22.86
PPD (mm)	2.69 ± 0.36	2.77 ± 0.65 *	0.05	2.93
CAL (mm)	3.02 ± 0.68	3.11 ± 0.68 *	0.05	2.94
IL-17 (pg/mL)	0.18 ± 0.03	0.21 ± 0.04 *	0.01 *	16.21

* *p* < 0.05 significant compared between the group-I with group-II before treatment.

**Table 4:** Comparison of periodontal and biochemical parameters between the groups one month after NSPT.

Observations	Group-I (Mean ± SD)	Group-II (Mean ± SD)	*p* Values	Percentage Difference (%)
Fasting blood glucose level (mg/dL)	113.25 ± 5.58	142.85 ± 6.26 *	0.001	23.12
HbA1c (%)	5.55 ± 0.26	6.61 ± 0.20 *	0.001	17.43
PI	0.86 ± 0.19	0.92 ± 0.39 *	0.05	6.74
GI	0.85 ± 0.25	1.10 ± 0.36 *	0.04	25.64
PPD (mm)	2.13 ± 0.27	2.42 ± 0.65 *	0.05	12.75
CAL (mm)	2.65 ± 0.59	2.60 ± 0.72 *	0.05	1.90
IL-17 (pg/mL)	0.16 ± 0.02	0.16 ± 0.03	0.99	6.45

* *p* < 0.05 significant compared between the group-I with group-II after treatment.
